# Does justice matter in voice? Inclusive leadership and employee voice: the moderating role of organizational justice perception

**DOI:** 10.3389/fpsyg.2023.1313922

**Published:** 2023-12-11

**Authors:** Lei Qi, Yuping Xu, Bing Liu

**Affiliations:** ^1^School of Business Administration, Shandong University of Finance and Economics, Jinan, China; ^2^School of Management, Shandong University, Jinan, China

**Keywords:** promotive voice, prohibitive voice, inclusive leadership, distributive justice, procedural justice, interactional justice

## Abstract

**Introduction:**

As a distinctive form of relational leadership, the impact of inclusive leadership on employee work behavior has been widely considered by scholars. The purpose of this study was to examine the positive effects of inclusive leadership on employee voice (promotive voice and prohibitive voice), and the moderating role of organizational justice perception (distributive justice, procedural justice, and interactional justice) on such positive effects.

**Methods:**

Based on social exchange theory, this study used a multi-wave and multi-source survey to obtain data from 258 subordinates and 52 team leaders from construction companies located in China.

**Results:**

The results showed that inclusive leadership was positively related to promotive voice and prohibitive voice. Distributive justice and interactional justice would strengthen the positive relationship between inclusive leadership and promotive voice. Distributive justice, procedural justice, and interactional justice would strengthen the positive relationship between inclusive leadership and prohibitive voice.

**Discussion:**

First, this study examined the impact of inclusive leadership on employee voice, emphasized the importance of inclusive leadership as the core of relational leadership, and provided guidance for improving employee promotive and prohibitive voice. Second, this study conceptualized organizational justice perception as distributive justice, procedural justice, and interactional justice, comprehensively considered the impact of the three dimensions of justice perception on the effectiveness of inclusive leadership, and provided specific reference for employee management practice from the perspective of justice.

## 1 Introduction

Scholars defined behaviors of providing suggestions as voice, which is intentionally expressing relevant ideas, thoughts, information, opinions, and concerns about possible work-related improvements (Van Dyne and LePine, 1998; Van Dyne et al., 2003). Voice can be beneficial in the workplace (Li and Tangirala, 2021). For instance, voice may promote higher performance (Mackenzie et al., 2011; Lam and Mayer, 2014) and greater management innovation (Guzman and Espejo, 2019) at the unit level, better team decision (Dooley and Fryxell, 1999) and more team productivity/safety performance gains (Li et al., 2017) at the team level, more work engagement (Alang et al., 2020) and higher employee performance evaluation (Howell et al., 2015) at the individual level. Given these benefits of voice, a growing number of studies explored the factors that foster or inhibit voice (Chamberlin et al., 2017).

Chamberlin, Newton, and LePine’s meta-analysis concluded that leader behavior is considered as a key antecedent of subordinate voice “because a leader can influence workplace norms regarding voice and directly encourage or hinder employee voice” (Chamberlin et al., 2017). Employees are highly attuned to the behavior of leaders and examine leader actions for information about what is expected and accepted (Tyler and Lind, 1992). Previous research verified that if a leader takes an authoritarian, unsupportive, or defensive stance, team members are more likely to feel that speaking up is unsafe (Nembhard and Edmondson, 2006; Li and Sun, 2015). In contrast, if a leader is democratic, supportive, open, and welcomes questions and challenges, team members are likely to feel greater psychological safety in their interactions with each other (Nembhard and Edmondson, 2006; Morrison, 2011). Inclusive leadership, as a special form of relational leadership (Chen and Cheng, 2021), emphasizes the two-way interaction and interdependence between leaders and employees and refers to the open, approachable, and tolerant style shown by leaders in their interaction with employees (Carmeli et al., 2010). Different from other leadership styles (e.g., transformational leadership, charismatic leadership, strategic leadership, and servant leadership), inclusive leadership pays more attention to supporting employees’ needs and expectations and tolerating failures and mistakes and tends to provide employees with opportunities to exercise their abilities and express their ideas (Ye et al., 2019). In an increasingly changing and complex organizational environment, inclusive leadership is particularly important (Guo et al., 2022). However, few studies have explored the relationship between inclusive leadership and subordinate voice (Qi and Liu, 2017; Guo et al., 2022; Jiang et al., 2022). Moreover, the boundary conditions of the effect of leader inclusiveness on subordinate voice remain unclear.

Previous research indicated that it is important to define a boundary condition under which leader influence becomes more or less effective (Guo et al., 2022). Employees’ voice is not only the results of leaders’ behavior and leadership (Ehrhart, 2004; Abd El Majid and Cohen, 2015; Abu Nasra and Heilbrunn, 2016), but also the reaction of organizational contextual factors (Wong et al., 2006; Hoffman et al., 2007; Lavelle, 2010). An important characteristic of inclusive leadership is the emphasis on justice and fairness, which promotes proactive behaviors by providing employees with fair opportunities to meet their needs for a sense of belonging and value (Guo et al., 2022; Santos et al., 2022). Nonetheless, organizational justice, as an important organizational situational factor, its impact on employee behavior cannot be overlooked as well (Shoaib and Baruch, 2017; Abuelhassan and AlGassim, 2022). If employees perceive low organizational justice, they will respond with negative strategies even if inclusive leaders strive to create a fair work environment for them. Social exchange theory provides a powerful explanation for research in the field of organizational justice, pointing out that when organizations give employees higher perceptions of justice, employees will return to the organization for the principle of reciprocity (Farid et al., 2023). Following this logic, organizational justice affects the relationship between inclusive leadership and employee voice, but the research in this area has been overlooked, creating a significant research gap. According to Cropanzano et al. (2007), we divide organizational justice into three dimensions: distributive justice, procedural justice, and interactional justice, which is one of the most common divisions. Distributive justice refers to employee assessments of the fairness of rewards they receive due to their contributions at work (Greenberg, 1990). Procedural justice refers to employees’ assessments of the fairness of the organization’s decision process (Lind and Tyler, 1988; Niehoff and Moorman, 1993). Interactional justice refers to the perception of the quality of the interpersonal treatment received during the decision-making procedures in the organization (Bies, 1986). According to Adams (1963), when one perceives the inequity compared with others, it’s likely that one will adjust the unpleasant status (Greenberg, 1990) and translate it into negative actions (Walster and Berscheid, 1973; Frey et al., 2013), such as keeping silence (Pinder and Harlos, 2001).

**Figure 1:**
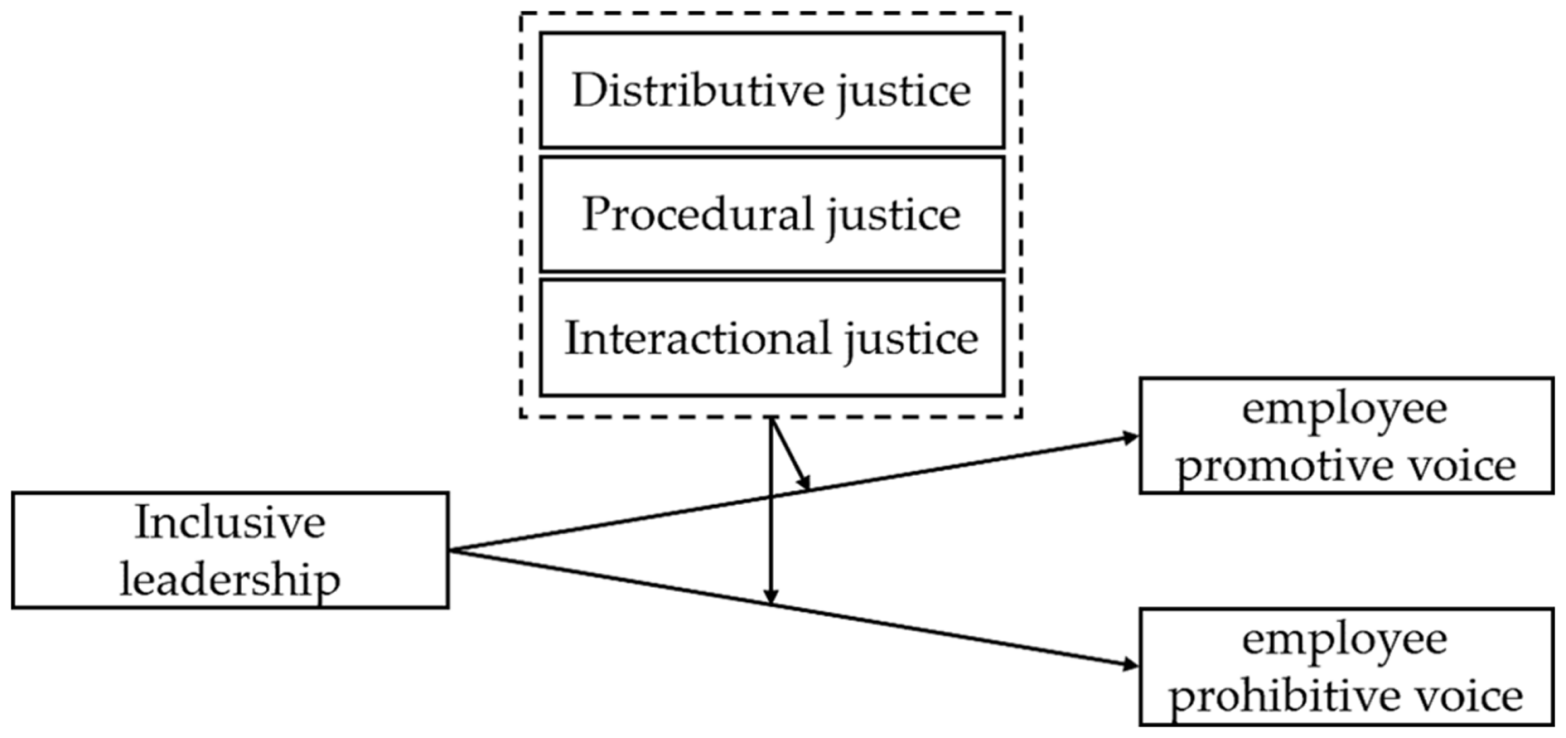
Conceptual model.

Based on the above analysis and the theoretical foundation of social exchange theory, the current study considers employee perception of workplace distributive justice, procedural justice, and interactional justice as moderating variables to examine the boundary conditions of inclusive leadership on subordinate voice. This research contributes to the extant literature in two ways. On the one hand, we broaden the understanding of inclusive leadership as a predictor of voice and that it responds to the call for more studies exploring issues concerning promotive and prohibitive voice (Chamberlin et al., 2017). On the other hand, our findings on the moderation role of organizational justice perception, enrich the knowledge on the contextual effectiveness of inclusive leadership. Figure 1 shows the conceptual model.

## 2 Theoretical background and hypotheses

### 2.1 Theoretical background

Based on social exchange theory, individuals will engage in and maintain exchange relationships with others with the expectation of getting returns, and choose a positive or negative response based on the principle of reciprocity (Gouldner, 1960). Social exchange theory further posits that the extent to which individuals choose positive strategies or negative strategies in return depends on whether they perceive reciprocity in the exchange process (Homans, 1958).

Exchange relationships are prevalent in organizations between employees and leaders (Settoon et al., 1996; Cropanzano and Mitchell, 2005). Inclusive leadership, as the core of relational leadership, focuses on building good interactions with employees, which itself a concrete manifestation of exchange relationships (Carmeli et al., 2010; Duc and Tho, 2023). The openness, accessibility, and effectiveness of inclusive leadership provide employees with the necessary resources to support and demonstrate recognition and affirmation of their value, which promotes employees to take positive strategies to reciprocate and facilitate the occurrence of employee voice (Qi and Liu, 2017). Specifically, employees may engage in more promotive and prohibitive voices. Promotive voice refers to employees actively putting forward new suggestions to improve the operation of the organization (Liang et al., 2012). Inclusive leaders’ recognition and appreciation of employees make employees feel that their leaders value their opinions, which promotes their promotive voice (Guo et al., 2022). Prohibitive advice refers to employees actively pointing out potential problems to prevent damage to organizational development (Liang et al., 2012). Inclusive leaders’ acceptance and tolerance of employees reduces employees’ perception of risk, which promotes their prohibitive advice (Wei et al., 2015).

However, organizational justice affects employees’ perceptions of reciprocity (Farid et al., 2023), that is, the impact of inclusive leadership on employee voice will be influenced by the extent of organizational justice. According to the classification standard of Cropanzano et al. (2007), we considered three dimensions of organizational justice (i.e., distributive justice, procedural justice, and interactional justice). Since distributive justice, procedural justice, and interactional justice have different emphases (Cropanzano et al., 2007), they may have different effects on the relationship between inclusive leadership and employee voice. We further elaborate on this point in the hypotheses section.

### 2.2 Research hypotheses

#### 2.2.1 Inclusive leadership and subordinate voice

Voice as employee openly communicate with either their colleagues or superiors about workplace matters and propose constructive suggestions or concerns even when others disagree (Premeaux and Bedeian, 2003; Van Dyne et al., 2003; Tangirala and Ramanujam, 2008), which will not happen in a vacuum, and the key triggering components involve the motives, individual, and situational factors (Morrison, 2011). To capture the various forms of voice occurring in practice, we follow Liang et al. (2012) to classify employee voice as promotive voice and prohibitive voice. The former refers to new ideas and suggestions for improving the work unit or organization, and the latter involves concerns about harmful work-related practices, incidents, or behavior (Liang et al., 2012). Among a variety of contextual factors (e.g., leader personality, relationship with supervisor, and leadership style) that have been proven to influence employee voice (Detert and Burris, 2007; Walumbwa and Schaubroeck, 2009; Morrison, 2011; Ng and Feldman, 2011; Avey et al., 2012; Hsiung, 2012; Li and Sun, 2015; Lee et al., 2017; Lin et al., 2017; Chen et al., 2018; Liu et al., 2019), one of the most important sources of cues about whether it is worthwhile and safe to voice is the behavior of one’s immediate supervisor (Detert and Burris, 2007; Morrison, 2011; Li and Sun, 2015). Employees who perceived more safety and less workplace stressors are more likely to speak up (Morrison, 2011; Ng and Feldman, 2011; Hsiung, 2012; Wei et al., 2015).

Inclusive leaders usually exhibit words and deeds that invite and appreciate followers’ contributions and actively establish a two-way relationship with subordinates based on respect, recognition, feedback, and responsibility (Nembhard and Edmondson, 2006; Hollander, 2012; Gallegos, 2013). According to the social exchange theory and the norm of reciprocity (Wayne et al., 1997), individual reciprocity is realized through mutual exchange and help, and leaders influence employee behavior through the social exchange process (Cropanzano and Mitchell, 2005). Inclusive leaders willing to build interactional relationships with subordinates (Hollander, 2012), pay more attention to them, acknowledge their contributions, and provide necessary needs to stimulate their potential (Randel et al., 2018). Thus, subordinates are likely to show corresponding positive job attitudes and behaviors such as voice as a return.

Inclusive leadership has a positive effect on employee prohibitive voice. First, prohibitive voice featured by challenge-oriented and potentially risky (Liu et al., 2010) challenge organizational status as well as others especially their superiors’ authority (Graham and Van Dyne, 2006), thus sometimes may impede one’s development or cause interpersonal conflict (Adler and Kwon, 2002; Milliken et al., 2003; Detert and Burris, 2007). Given the circumstance, employees will weigh the costs and safety before they choose to reflect concerns on undesirable practices in work units (Chamberlin et al., 2017). However, inclusive leaders who are always democratic and supportive show openness and respect to followers and attach great importance to employees’ thoughts and feelings in the workplace (Nembhard and Edmondson, 2006). It is expected that the high-quality relationship between leader and employee will motivate employees to take risks in showing their concerns about the organization (Carnevale et al., 2017). This is conducive to enhancing trust and psychological safety (Nembhard and Edmondson, 2006; Carmeli et al., 2010; Hirak et al., 2012; Javed et al., 2017), which are positively related to employee voice (Brockner et al., 2001; Detert and Burris, 2007; Walumbwa and Schaubroeck, 2009). Second, according to Nembhard and Edmondson’s research (Nembhard and Edmondson, 2006), leader inclusiveness not only accepts and encourages team members’ heterogeneity and diversity (Hollander, 2012; Jin et al., 2017), but also promotes followers’ perception of belongingness and helps them get the best of their uniqueness to capture unite success (Randel et al., 2018). Prohibitive voice involves characteristics of creativity and initiative (Ng and Feldman, 2011), which connect with the full use of one’s uniqueness (Dollinger, 2003; Chu and Lin, 2013). At the same time, inclusive leadership also plays an important role in fostering inclusive cultures (Ainscow and Sandill, 2010), in which employees do not need to tread on eggs or worry about negative treatment. Thus, it’s highly likely that they will give prohibitive advice. We thus hypothesize:

*Hypothesis 1a*. Inclusive leadership will be positively related to employee prohibitive voice.

Employee promotive voice will also be influenced by inclusive leadership. First, the openness, accessibility, and availability manifested by leader inclusiveness, send a signal to subordinates that their leader is competent and willing to build close interpersonal relationships and provide needs if necessary (Carmeli et al., 2010). Inclusive leaders ask subordinates for ideas about the improvement of unit work and can take timely action on the issues identified by employees (Nembhard and Edmondson, 2006). The more efficacy of voice an employee perceives, the more likely for one to do such behavior (Morrison, 2011). Second, inclusive leaders cultivate a harmonious context in which employees are welcome and have the opportunity to exchange ideas, provide constructive suggestions, and be involved in the unit business (Carmeli et al., 2010). Under the inclusive and participatory circumstance, considering the social exchange theory and the norm of reciprocity (Wayne et al., 1997), employees will do extra-role behaviors such as promotive voice in return (Liang et al., 2012; Harris et al., 2014; Chenevert et al., 2015). Therefore, we contend that inclusive leadership is positively linked to both employee promotive voice and prohibitive voice. We thus hypothesize:

*Hypothesis 1b*. Inclusive leadership will be positively related to employee promotive voice.

#### 2.2.2 Organizational justice perception as moderator

##### 2.2.2.1 The moderating role of distributive justice

Based on equity theory (Adams, 1963) and social exchange theory (Emerson, 1976), employees will weigh their engagement and output during the workplace compared with other colleagues (Cohen-Charash and Spector, 2001; Colquitt et al., 2001), and the results will further affect their initiative and work behaviors (Adams, 1965; Asghar Pourezzat and Zeinali Someh, 2009). High perceptions of workplace distributive justice will enhance the positive relationship between inclusive leadership and employee voice.

First, based on social exchange theory (Emerson, 1976), whether employees choose to speak up or not mainly lies on the expected results of voice (Detert and Burris, 2007). With high distributive justice, inclusive leaders’ support and help proved to work and employees get satisfactory rewards. Employees are encouraged and feel obliged to express a promotive voice in return (Wayne et al., 1997). Second, the high perception of distributive justice convinces employees that the organization will distribute according to established principles (Colquitt et al., 2001). This sense can provide messages that inclusive leaders are worthy of trust (Hopkins and Weathington, 2006), employee’s psychological safety is improved and thus will be more likely to provide a prohibitive voice (Liang et al., 2012).

On the contrary, when employees perceive low workplace distributive justice, they will distort their perceptions of their leaders and organization (Hartman et al., 1999; Thierry, 2002), the positive effect of inclusive leadership on employee voice will be weak. Low perceptions of distributive justice make employees believe that their contributions and performance are not reasonably considered by their leaders (Adams, 1963) and thus weaken subordinates’ trust to respect and support leaders’ inclusiveness (Lipponen et al., 2004). According to social exchange theory, now that their value is not accepted or measured by the organization, employees can hardly get satisfactory remuneration, they are not willing to put in a lot of effort as before (Nahrgang et al., 2011). As a result, subordinates will seldom or even refuse to propose constructive advice and promotive voice (Holland et al., 2016; Kwon et al., 2016). In addition, although inclusive leaders are accessible and provide opportunities for subordinates to speak up (Nembhard and Edmondson, 2006; Hollander, 2012), the distorted cognition caused by the unfairness (Adams, 1963) contributed to the risk and suspicion, which also impede them from showing their prohibitive voice. According to the above, the hypothesis is proposed:

*Hypothesis 2*. Distributive justice moderates the relationship between inclusive leadership and employee (a) promotive voice and (b) prohibitive voice, such that the relationships are more positive at higher (versus lower) levels of distributive justice.

##### 2.2.2.2 The moderating role of procedural justice

Thibaut and Walker (1975) brought procedural research into justice theory and proposed procedural justice, which is defined as employees’ assessments of the fairness of the organization’s decision process (Lind and Tyler, 1988; Niehoff and Moorman, 1993). Engagement, explanation, and clarity of expectations are three criteria of procedural justice in a business setting (Kim and Mauborgne, 1997), which has already included the components that affecting employee voice. We proposed that procedural justice can influence the relationship between inclusive leadership and voice.

Previous studies on procedural justice perceptions argue that, if the implementation of organizational decision procedures is just and unbiased, signals of goodwill from inclusive leaders are easier to recognize because employees will see their organization as both reliable and trustworthy (Hon and Lu, 2010; Tremblay et al., 2010; Guh et al., 2013; Agarwal, 2014). Then, the inclusive climate in the team will be more effective and thus motivate employees to conduct prohibitive voice. In addition, in the inclusive climate, heterogeneity and the presence of different voices are allowed (Ainscow and Sandill, 2010). At the same time, the organization, furnished with fair procedures, will consider employees’ opinions when making decisions (Konovsky, 2000; Pichler et al., 2015). Based on organizational justice theory (Colquitt et al., 2001), employees’ control needs are satisfied and the efficacy of voice is improved (Morrison, 2011). Thus, employees are more likely to provide promotive voice.

On the contrary, when procedural justice is low, the positive relationship between inclusive leadership and voice will be weakened. First, compared with the unfairness of distribution, procedural unfairness further increases the team members’ doubts about the rationality of the performance results (Thibaut and Walker, 1975). In this case, even if leaders are open and available, employees will keep silent because they are not allowed to participate in the decision process (Niehoff and Moorman, 1993) and their voice is useless to some extent (Morrison, 2011). In addition, people collect information about their social identity by analyzing the fairness of the program (Lind and Tyler, 1988). Low level of procedural justice leads employees to believe that the organization does not respect them as valued members of the group (Lind, 2001), which in turn restrains the unique value of employees and from putting forward a prohibitive voice. Hence, the following hypothesis is suggested.

*Hypothesis 3*. Procedural justice moderates the relationship between inclusive leadership and employee (a) promotive voice and (b) prohibitive voice, such that the relationships are more positive at higher (versus lower) levels of procedural justice.

##### 2.2.2.3 The moderating role of interactional justice

Interactional justice refers to the perception of the quality of the interpersonal treatment received during the decision-making procedures in the organization (Bies, 1986), which is mainly related to social exchange between employees and their supervisors (Cropanzano et al., 2001). The quality of the social exchange relationship will affect the play of leadership influence and employees’ reciprocal behaviors (Cropanzano and Mitchell, 2005). We proposed that interactional justice can influence the relationship between inclusive leadership and voice.

In the context of high interactional justice, inclusive leaders have a stronger positive relationship with employee promotive voice, because employees are more likely to grasp access to advice. In addition, a high level of interactional justice promotes employees’ perception of the quality of their relationship with leaders (Colquitt et al., 2001), and leads to trust in leaders (Ambrose and Schminke, 2003). At this time, the role of inclusive leadership is effectively played, and the openness, accessibility, and support from leaders further enhance the psychological security of employees (Carmeli et al., 2010; Javed et al., 2017) and facilitate prohibitive voice (Detert and Burris, 2007).

On the contrary, low interactional justice makes employees feel their needs were not considered and they are treated badly (Colquitt et al., 2001). The experience of contacting superiors is so unpleasant that employees will ignore the opportunity and channel the inclusive leaders provide. Additionally, since employees’ self-respect and control needs are not met, employees will not do extra-role behavior (e.g., promotive voice) in return (Nohe and Hertel, 2017). Moreover, as the prohibitive voice involves authority challenge as well as interpersonal risk (Graham and Van Dyne, 2006; Liu et al., 2010), it may spoil the image and cause interactional conflict (Adler and Kwon, 2002; Milliken et al., 2003; Detert and Burris, 2007). Under low interactional justice circumstances, employees may have more strained relationships with their bosses and co-workers (He et al., 2017). Even if leaders are open and available, subordinates still see prohibitive voice as unpleasant behavior and question the value of voice. Thus, the positive relationship between inclusive leadership and voice is weaker. The hypothesis is proposed:

*Hypothesis 4*. Interactional justice moderates the relationship between inclusive leadership and employee (a) promotive voice and (b) prohibitive voice, such that the relationships are more positive at higher (versus lower) levels of interactional justice.

## 3 Methods

### 3.1 Participants and procedure

To test the conceptual model, we collected data from subordinates and their direct supervisors in project teams of Chinese construction companies. The project teams of Chinese construction companies are ideal samples for three reasons. First, the team as the basic unit of task execution has become the foundation stone of modern organization. The project team is the typical operation unit in the construction industry, providing a great platform to observe team factors. Second, influenced by factors such as project size and cycle length, different project teams in the construction industry are representative as they have different personnel composition and team status. Third, the senior managers of the target companies were the EMBA students who had great interest in our research. We adopted the questionnaire survey and set up a research team to communicate with senior managers, human resource directors, and project team leaders of relevant enterprises. In our initial contact with the participants, we provided a general overview of the research but did not disclose any specific research hypotheses to them.

To minimize the common method variance, multi-time and multi-source data were collected. We prepared two types of questionnaires and collected two waves of data with a three-month time lag. “Team supervisor- subordinates” mutual evaluation method is used. At Time 1, employees assessed inclusive leadership, authentic leadership, humble leadership, and organizational justice and provided personal information. At Time 2, supervisors assessed employee voice and provided personal information. After supervisors completed their evaluations, we formulated codes for the questionnaire to ensure that the supervisor data matched those of their subordinates. Each participant was compensated with 5–10 RMB and their personal information was promised to be kept to maximize the response rate.

We distributed 350 questionnaires to 60 project teams. After matching the questionnaires of supervisors and subordinates, we received 322 responses from 53 groups (92.0% response rate). Samples with missing or invalid information on core variables were excluded. As a result, 258 effective questionnaires (73.7% response rate) from 52 groups remained for our analyses. Of the 258 subordinates sampled, 73.6% were men, the average age was 30.09, 76.4% had received post-graduate degrees, and the average working experience was 7.32 years.

### 3.2 Measures

#### 3.2.1 Inclusive leadership

We measured inclusive leadership with a sixteen-item scale constructed by Hollander (Hollander, 2012). Respondents reported the respect, understanding, and feedback that employees perceived from their leaders. Sample items include “My leader often asks me for ideas about my work,” “My leader can take timely action on the issues identified by employees” and “My leader always criticizes me in front of others when things go wrong (R)” (1 = strongly disagree to 5 = strongly agree). The Cronbach alpha for this measure was 0.90.

#### 3.2.2 Organizational justice perception

A twenty-item scale developed by Niehoff and Moorman was used to measure organizational justice perception (Niehoff and Moorman, 1993). Of the 20 items, 5 items measured distributive justice (e.g., “I consider my workload to be quite fair”), 6 items measured procedural justice (e.g., “Job decisions are made by the general manager in an unbiased manner.”), 9 items measured interactional justice (e.g., “When decisions are made about my job, the general manager treats me with kindness and consideration”). The Cronbach alpha of procedural justice, interactional justice, and distributive justice were 0.91, 0.95, and 0.91, respectively.

#### 3.2.3 Voice

We used Liang et al.’s (2012) 10-item scale to assess voice. Of the 10 items, 5 items measured promotive voice (e.g., “Proactively develop and make suggestions for issues that may influence the group”), 5 items measured prohibitive voice (e.g., “Advise other colleagues against undesirable behaviors that would hamper job performance”). The Cronbach alpha of promotive voice and prohibitive voice were 0.88 and 0.83, respectively.

#### 3.2.4 Control variables

We included employees’ age, gender, education level, and job tenure as control variables because of their potential impact on employee voice. Besides, we also controlled for prior voice antecedents: authentic leadership and humble leadership. Given that these two different types of leadership may also affect employees’ voice (Wong et al., 2010; Hsiung, 2012; Lin et al., 2017). A sixteen-item scale developed by Neider and Schriesheims was used to measure authentic leadership (Neider and Schriesheim, 2011). Sample items include (e.g., “My leader clearly states what he/she means,” “My leader shows consistency between his/her beliefs and actions,” “My leader asks for ideas that challenge his/her core beliefs,” and “My leader describes accurately the way that others view his/her abilities”) (1 = strongly disagree to 5 = strongly agree, *α* = 0.94). We used Owens et al.’s (2013) 9-item scale to assess humble leadership. Sample items include “This person often compliments others on their strengths” and “This person actively seeks feedback, even if it is critical” (1 = strongly disagree to 5 = strongly agree, *α* = 0.91).

## 4 Results

### 4.1 Descriptive statistics

Descriptive statistics and correlations are presented in Table 1. As shown in the table, inclusive leadership was positively related to both promotive voice (*r* = 0.206, *p* < 0.01) and prohibitive voice (*r* = 0.180, *p* < 0.01). In addition, authentic leadership (*r* = 0.134, *p* < 0.05, for prohibitive voice) and humble leadership (*r* = 0.137, *p* < 0.05; *r* = 0.128, *p* < 0.05, respectively) were also related to voice. These results were consistent with and provided initial support to our hypotheses.

**Table 1:** Means, standard deviation, and correlations between study variables.

Variables	1	2	3	4	5	6	7	8	9	10	11	12
1. Gender ^1^												
2. Age	−0.003											
3. Tenure	−0.061	0.841**										
4. Education ^2^	−0.167**	−0.267**	−0.185**									
5. Authentic leadership	0.103	−0.019	0.009	−0.001	(0.94)							
6. Humble leadership	0.102	0.016	0.054	0.028	0.856**	(0.91)						
7. Inclusive leadership	0.147*	0.017	0.011	−0.014	0.789**	0.747**	(0.90)					
8. Procedural justice	0.013	0.012	0.039	−0.023	0.818**	0.810**	0.716**	(0.91)				
9. Interactional justice	0.115	0.036	0.037	−0.010	0.767**	0.805**	0.741**	0.824**	(0.95)			
10. Distributive justice	0.075	−0.045	−0.052	−0.061	0.432**	0.498**	0.371**	0.495**	0.474**	(0.91)		
11. Promotive voice	−0.041	−0.078	−0.034	0.096	0.091	0.137*	0.206**	0.163**	0.152*	0.008	(0.88)	
12. Prohibitive voice	0.057	−0.137*	−0.087	0.052	0.134*	0.128*	0.180**	0.188**	0.159*	0.048	0.737**	(0.83)
Mean	1.267	30.089	7.325	3.787	4.009	4.105	4.086	4.040	4.108	3.681	4.005	4.019
S. D.	0.443	6.989	6.917	0.541	0.727	0.729	0.631	0.731	0.757	0.851	0.661	0.635

*N* = 258. *p ≤ 0.05. **p ≤ 0.01. Bracketed values on the diagonal are the Cronbach’s alpha value of each scale. ^1^ 1_male, 2_female; ^2^ 1_senior high school or below, 2_junior college, 3_college, 4_bachelor, 5_ postgraduate or above.

### 4.2 Harman’s one-factor test

Since the data on inclusive leadership, procedural justice, distributive justice, and interactional justice were collected from the same source, we conducted Harman’s one-factor test to evaluate common method variance (CMV). According to the results, the first factor explained only 35.21% of the variance, below the critical value of 40%. In addition, the one-factor model also showed poor fit (*χ*^2^ = 2625.227, df = 299, root mean square error of approximation (RMSEA) = 0.174, comparative fit index (CFI) = 0.508, Tucker–Lewis Index (TLI) = 0.465), which provided that CMV was not a significant problem in this research (Podsakoff et al., 2003).

### 4.3 Confirmatory factor analyses (CFA)

We used the same analytical strategies for CFA and hypothesis testing that we used in Study 1. The hypothesized six-factor model (i.e., inclusive leadership, procedural justice, distributive justice, interactional justice, promotive voice, and prohibitive voice) suggested a good fit to the data: *χ*^2^ = 661.745, df = 284, *χ*^2^/df = 2.290, RMSEA = 0.072, CFI = 0.920, TLI = 0.909. Results (see Table 2) showed that the hypothesized six-factor model was superior compared with other models. Therefore, the empirical distinctiveness among the variables of our research was confirmed.

**Table 2:** Confirmatory factor analysis results.

Model	χ^2^	*Df*	RMSEA	SRMR	CFI	TLI
Six-factor model (IL, PJ, IJ, DJ, PV, PRV)	661.745	284	0.072	0.059	0.920	0.909
Five-factor model (IL, PJ, IJ, DJ, PV + PRV)	726.774	289	0.077	0.058	0.907	0.896
Five-factor model (IL, PJ + IJ, DJ, PV, PRV)	781.508	289	0.081	0.060	0.896	0.883
Five-factor model (IL, PJ + DJ, IJ, PV, PRV)	1249.008	289	0.114	0.088	0.797	0.772
Five-factor model (IL, PJ, IJ + DJ, PV, PRV)	1282.554	289	0.116	0.112	0.790	0.764
One-factor model (IL + PJ + IJ + DJ + PV + PRV)	2625.227	299	0.174	0.179	0.508	0.465

*N* = 258. RMSEA, root mean square error of approximation; SRMR, standardized root mean square residual; TLI, Tucker-Lewis index; CFI, comparative fit index; IL, inclusive leadership; PJ, procedural justice; IJ, interactional justice; DJ, distributive justice; PV, promotive voice; PRV, prohibitive voice.

### 4.4 Hypothesis testing

As shown in Table 3, when we controlled employees’ gender, age, education, and tenure, inclusive leadership was positively related to (a) promotive voice (*β* = 0.226, *p* < 0.01, Model 2) and (b) prohibitive voice (*β* = 0.178, *p* < 0.01, Model 4). While the results in Table 4 showed that when we added authentic leadership and humble leadership as control variables, inclusive leadership was also positively related to (a) promotive voice (*β* = 0.373, *p* < 0.01, Model 2) and (b) prohibitive voice (*β* = 0.211, *p* < 0.05, Model 4). Moreover, compared with the two tables, the effects between inclusive leadership and employee voice were changed obviously, which partially proved that inclusive leadership influenced employees’ voice more efficiently after controlling the other two kinds of leadership. Thus, Hypothesis 1a and 1b were supported.

**Table 3:** Results of hierarchical regression analysis without two control variables.

Variables	Promotive voice		Prohibitive voice	
	Model 1	Model 2	Model 3	Model 4
Control variables				
Gender ^1^	−0.036	−0.084	0.096	0.058
Age	−0.013	−0.013	−0.020	−0.020
Tenure	0.009	0.008	0.010	0.009
Education ^2^	0.090	0.085	0.029	0.026
Independent variable				
Inclusive leadership		0.226**		0.178**
*R* ^2^	0.015	0.061	0.026	0.057
ΔR^2^	0.015	0.046	0.026	0.031
F	0.989	3.276**	1.686	3.020
ΔF	0.989	12.251**	1.686	8.165**

*N* = 258. *p ≤ 0.05. **p ≤ 0.01. ^1^ 1_male, 2_female; ^2^ 1_senior high school or below, 2_junior college, 3_college, 4_bachelor, 5_ postgraduate or above.

**Table 4:** Results of hierarchical regression analysis with two control variables.

Variables	Promotive voice		Prohibitive voice	
	Model 1	Model 2	Model 3	Model 4
Control variables				
Gender ^1^	−0.059	−0.089	0.074	0.057
Age	−0.012	−0.015	−0.019	−0.020
Tenure	0.006	0.009	0.008	0.010
Education ^2^	0.077	0.078	0.025	0.025
Authentic leadership	−0.088	−0.268*	0.066	−0.036
Humble leadership	0.200	0.113	0.049	0.000
Independent variable				
Inclusive leadership		0.373**		0.211*
*R* ^2^	0.036	0.081	0.042	0.057
ΔR^2^	0.036	0.044	0.042	0.015
F	1.582	3.142**	1.824	2.165*
ΔF	1.582	12.082**	1.824	4.080*

*N* = 258. *p ≤ 0.05. **p ≤ 0.01. ^1^ 1_male, 2_female; ^2^ 1_senior high school or below, 2_junior college, 3_college, 4_bachelor, 5_ postgraduate or above.

**Figure 2:**
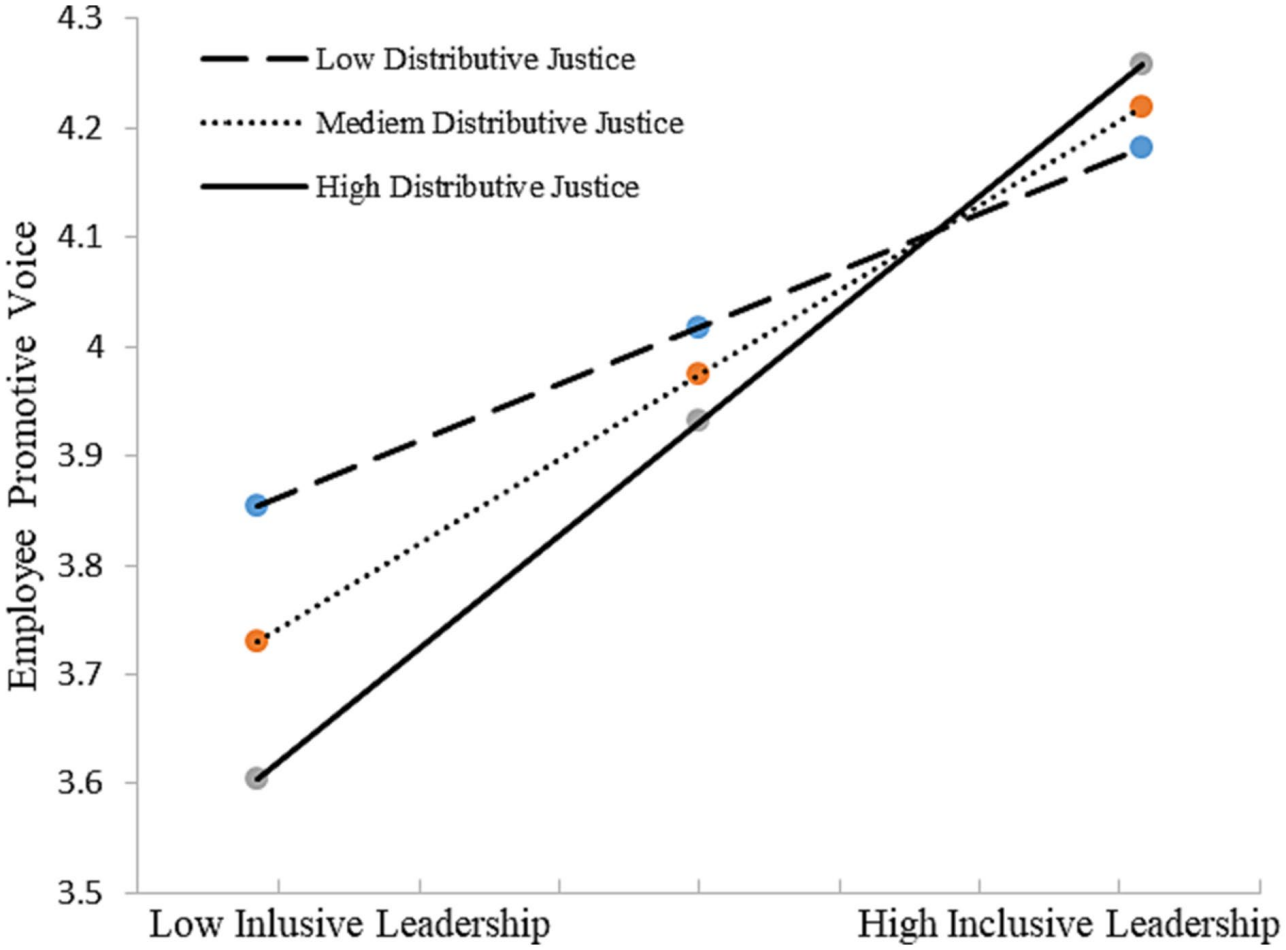
Interactive effects of inclusive leadership and distributive justice on promotive voice.

**Figure 3:**
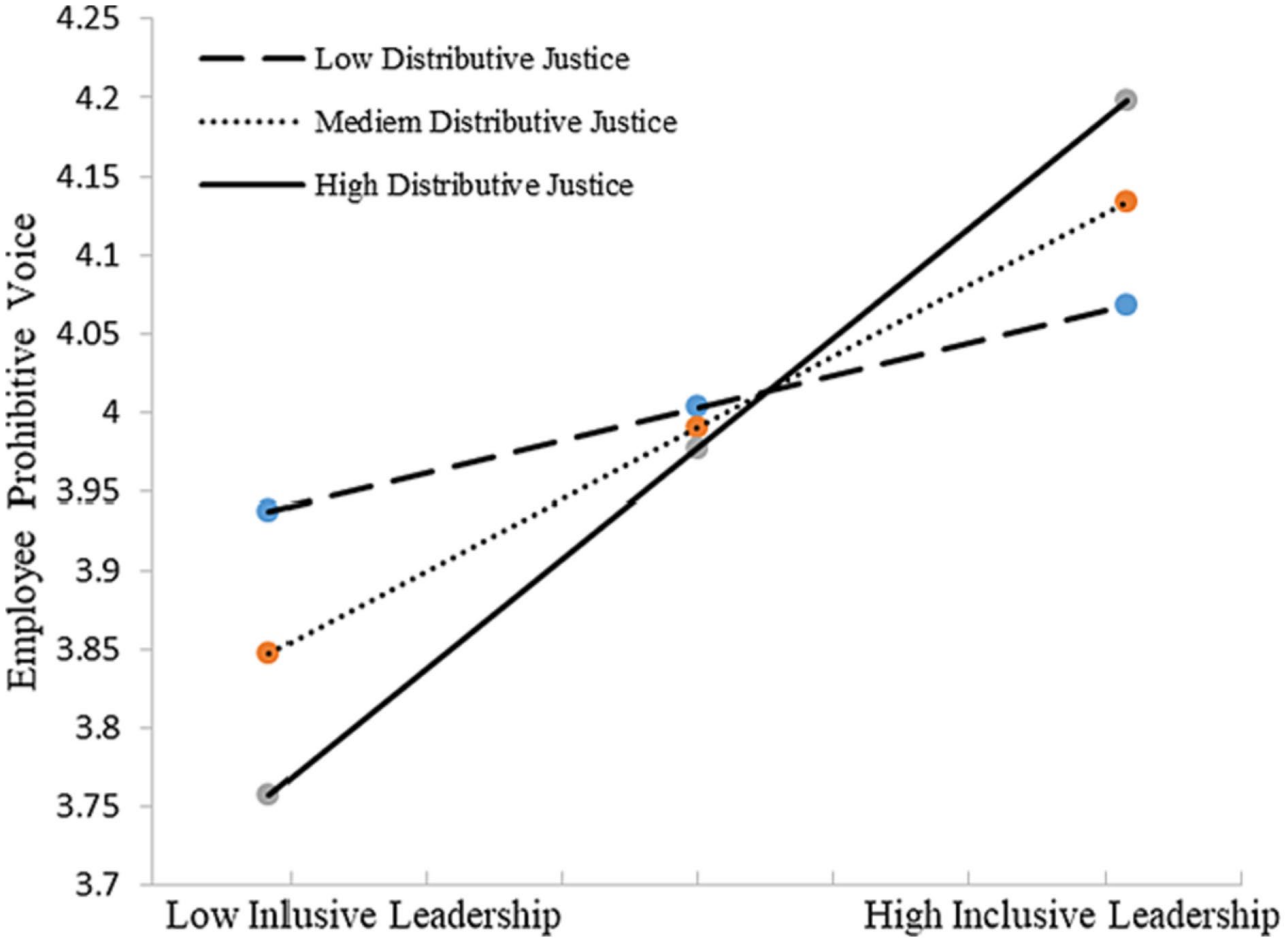
Interactive effects of inclusive leadership and distributive justice on prohibitive voice.

We conducted bootstrapping analyses (bootstrap = 5,000) to test our Hypothesis 2–4 with PROCESS macro in SPSS 20 (Hayes, 2017). Hypothesis 2–4 predicts that organizational justice perception [(a) distributive justice, (b) procedural justice, (c) interactional justice] moderate the positive relationship between inclusive leadership and employee voice. We first test Hypothesis 2, the moderation effect of distributing justice. As shown in Table 5, results suggested that the interaction terms were significant for both employee promotive voice (*β* = 0.151, *p* < 0.05) and prohibitive voice (*β* = 0.144, *p* < 0.05). Thus, H2 was supported. Figures 2, 3 depicts in more detail the nature of this moderation, showing that inclusive leadership had a more significantly positive effect on employee promotive and prohibitive voice among those who reported higher levels of distributed justice (*β* = 0.517, *p* < 0.01; *β* = 0.350, *p* < 0.01, respectively).

**Table 5:** Results of the moderating role of distributive justice.

Variables	Promotive voice			Prohibitive voice		
	*β*	SE	CI	*β*	SE	CI
Control variables						
Gender ^1^	−0.091	0.093	[−0.273, 0.091]	0.055	0.090	[−0.122, 0.233]
Age	−0.014	0.011	[−0.035, 0.007]	−0.020	0.011	[−0.041, 0.001]
Tenure	0.009	0.011	[−0.013, 0.030]	0.010	0.011	[−0.011, 0.031]
Education ^2^	0.060	0.078	[−0.094, 0.214]	0.013	0.076	[−0.138, 0.163]
Authentic leadership	−0.281*	0.118	[−0.514, −0.048]	−0.049	0.115	[−0.276, 0.178]
Humble leadership	0.176	0.115	[−0.050, 0.402]	0.040	0.112	[−0.180, 0.261]
Independent variable						
Inclusive leadership	0.389**	0.107	[0.179, 0.599]	0.227*	0.104	[0.022, 0.432]
Moderator						
Distributive Justice	−0.051	0.054	[−0.158, 0.056]	−0.015*	0.053	[−0.119, 0.090]
Interactive effect						
Inclusive leadership × Distributive justice	0.151*	0.071	[0.011, 0.290]	0.144*	0.069	[0.008, 0.280]
*R* ^2^	0.101**			0.074*		
ΔR^2^	0.016*			0.016*		
F	3.098			2.199		
ΔF	4.522			4.356		

*N* = 258. *p ≤ 0.05. **p ≤ 0.01. ^1^ 1_male, 2_female; ^2^ 1_senior high school or below, 2_junior college, 3_college, 4_bachelor, 5_ postgraduate or above.

**Figure 4:**
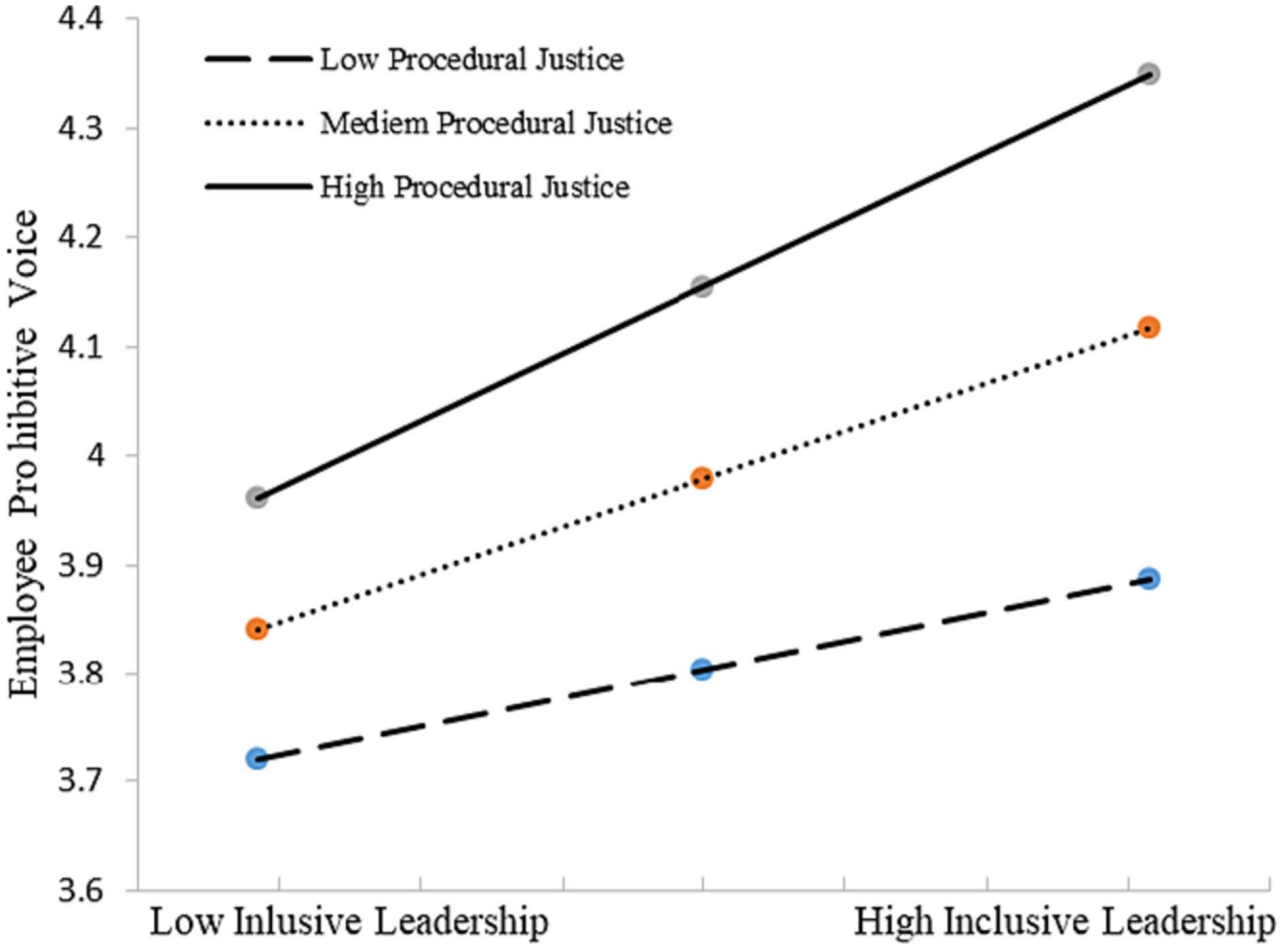
Interactive effects of inclusive leadership and procedural justice on prohibitive voice.

H3 proposes that procedural justice moderates the positive relationship between inclusive leadership and employee voice [(a) promotive voice and (b) prohibitive voice]. As shown in Table 6, the interaction was not significant in predicting employee promotive voice (*β* = 0.112, ns), but was significant in predicting employees’ prohibitive voice (*β* = 0.120, *p* < 0.05, *R*^2^ change = 0.014, *p* < 0.05). Thus, Hypothesis 3a was not supported, and 3b was supported. To illustrate the nature of the interaction effect, Aiken et al. (1991) procedure of computing slopes at one standard deviation above and below the mean of power distance was used to plot the interaction. Figure 4 presents the interaction patterns, the positive relationship between inclusive leadership and prohibitive voice is stronger when procedural justice is higher (*β* = 0.307, *p* < 0.05) than lower (*β* = 0.132, ns).

**Table 6:** Results of the moderating role of procedural justice.

Variables	Promotive voice			Prohibitive voice		
	*β*	SE	CI	*β*	SE	CI
Control variables						
Gender ^1^	−0.063	0.094	[−0.248, 0.123]	0.094	0.091	[−0.085, 0.273]
Age	−0.014	0.011	[−0.035, 0.007]	−0.020	0.010	[−0.040, 0.001]
Tenure	0.009	0.011	[−0.012, 0.030]	0.010	0.010	[−0.011, 0.030]
Education ^2^	0.075	0.078	[−0.079, 0.230]	0.025	0.076	[−0.124, 0.175]
Authentic leadership	−0.327**	0.125	[−0.574, −0.081]	−0.120	0.121	[−0.358, 0.118]
Humble leadership	0.054	0.116	[−0.175, 0.283]	−0.083	0.113	[−0.305, 0.139]
Independent variable						
Inclusive leadership	0.388**	0.110	[0.171, 0.604]	0.219*	0.106	[0.010, 0.428]
Moderator						
Procedural justice	0.177	0.105	[−0.029, 0.383]	0.240*	0.101	[0.041, 0.440]
Interactive effect						
Inclusive leadership × Procedural justice	0.112	0.063	[−0.012, 0.236]	0.120*	0.061	[0.00, 0.240]
*R* ^2^	0.101**			0.090**		
ΔR^2^	0.011			0.014*		
F	3.098			2.711		
ΔF	3.157			3.883		

*N* = 258. *p ≤ 0.05. **p ≤ 0.01. ^1^ 1_male, 2_female; ^2^ 1_senior high school or below, 2_junior college, 3_college, 4_bachelor, 5_ postgraduate or above.

**Figure 5:**
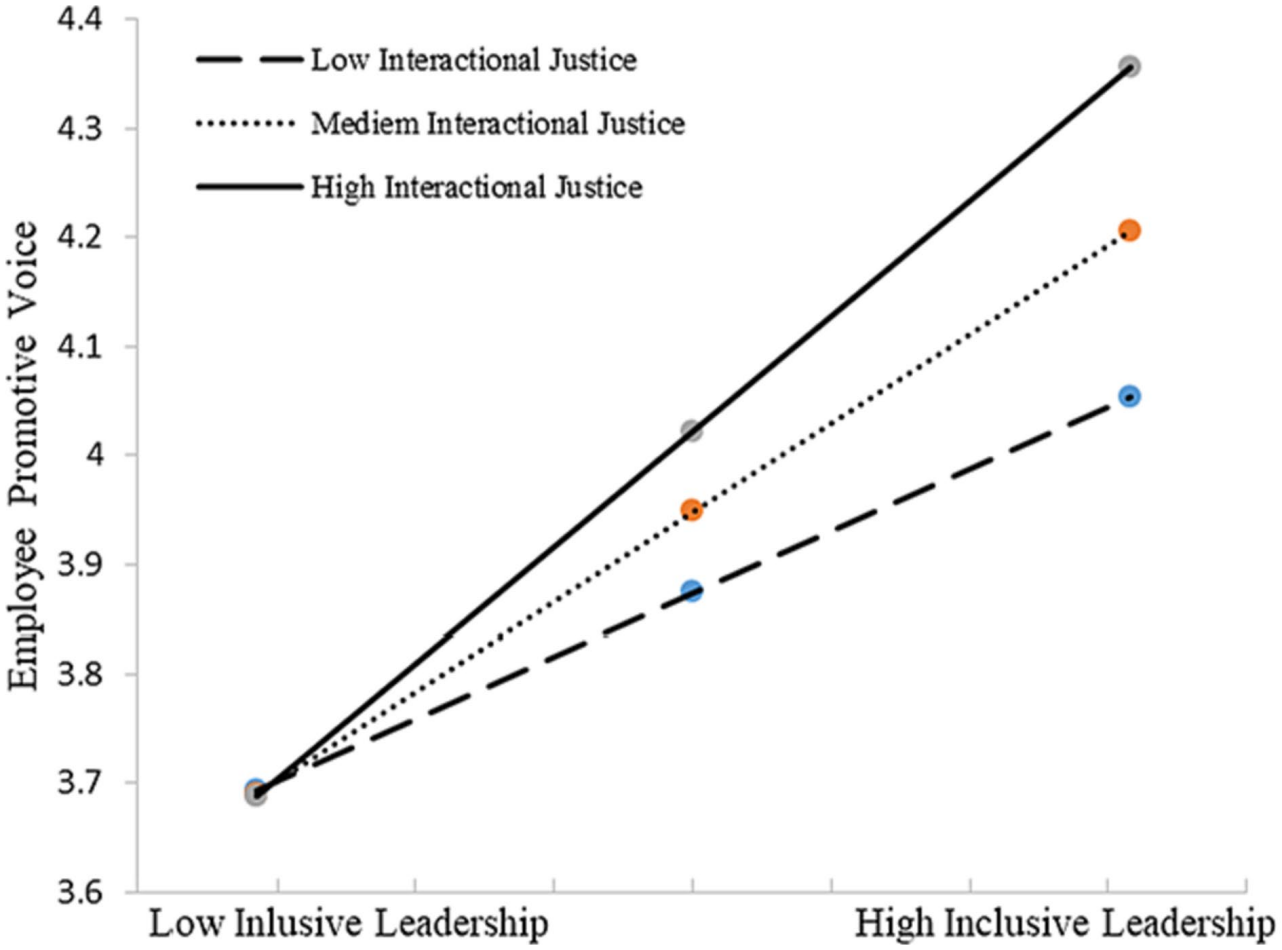
Interactive effects of inclusive leadership and interactional justice on promotive voice.

**Figure 6:**
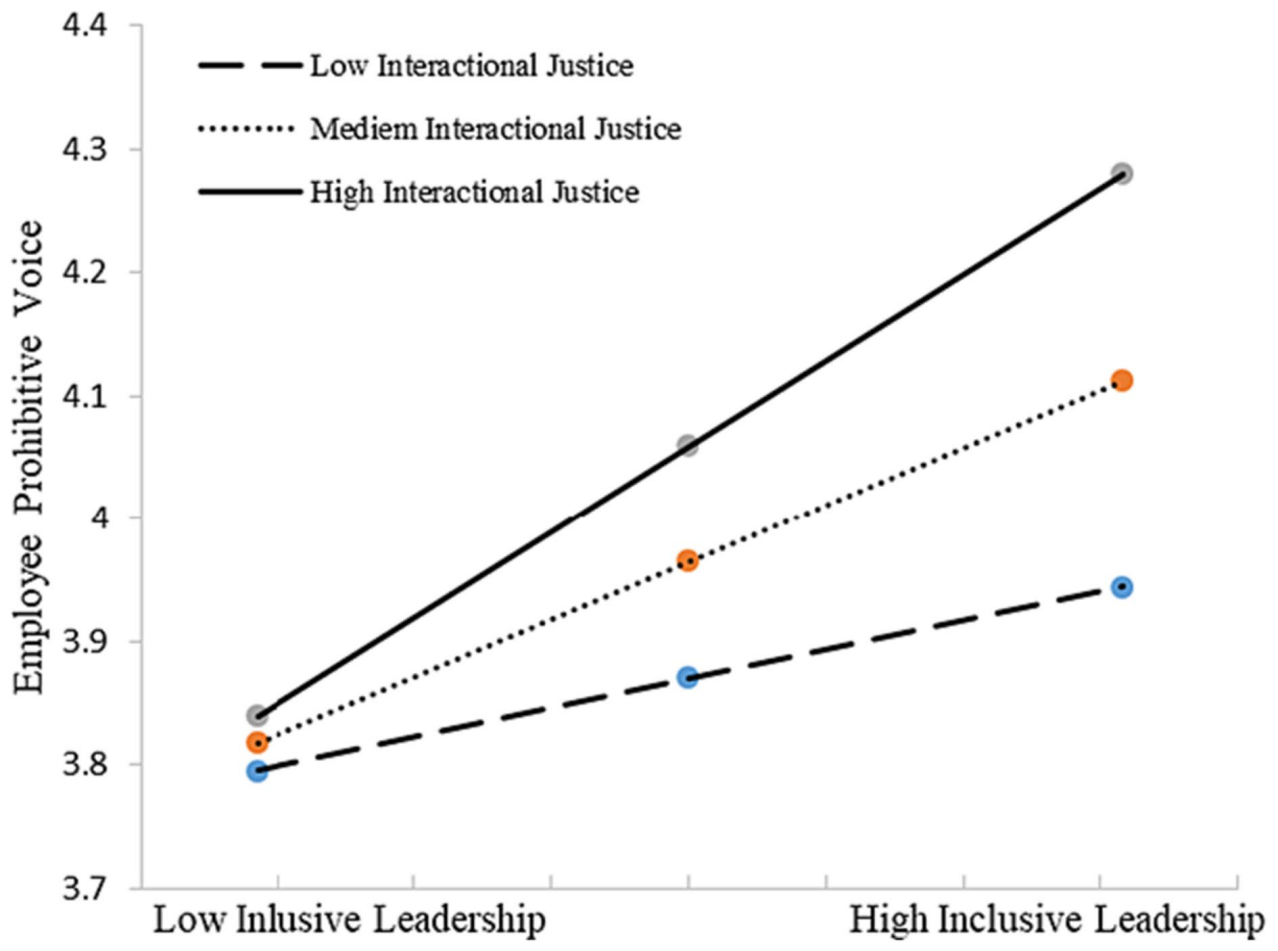
Interactive effects of inclusive leadership and interactional justice on prohibitive voice.

H4 predicts that interactional justice moderates the positive relationship between inclusive leadership and employee voice [(a) promotive voice and (b) prohibitive voice]. The results (see Table 7) for the interaction terms were significant for both employee promotive voice (*β* = 0.160, *p* < 0.01, *R*^2^ change = 0.025, *p* < 0.01) and prohibitive voice (*β* = 0.152, *p* < 0.05, *R*^2^ change = 0.024, *p* < 0.05). Therefore, H4 was supported. Figures 5, 6 provide additional evidence, which is consistent with our expectation: when the level of interactional justice was high, the effect of inclusive leadership on employee voice is higher [(a) promotive voice and (b) prohibitive voice] (*β* = 0.529, *p* < 0.01; *β* = 0.349, *p* < 0.01, respectively).

**Table 7:** Results of the moderating role of interactional justice.

Variables	Promotive voice			Prohibitive voice		
	*β*	SE	CI	*β*	SE	CI
Control variables						
Gender ^1^	−0.094	0.092	[−0.275, 0.088]	0.053	0.090	[−0.124, 0.229]
Age	−0.014	0.011	[−0.035, 0.007]	−0.020	0.011	[−0.041, 0.001]
Tenure	0.009	0.011	[−0.012, 0.030]	0.010	0.010	[−0.011, 0.031]
Education ^2^	0.064	0.078	[−0.089, 0.217]	0.013	0.076	[−0.138, 0.162]
Authentic leadership	−0.278*	0.119	[−0.512, −0.045]	−0.051	0.115	[−0.278, 0.176]
Humble leadership	0.087	0.119	[−0.147, 0.322]	−0.041	0.116	[−0.269, 0.187]
Independent variable						
Inclusive leadership	0.407**	0.112	[0.186, 0.628]	0.233*	0.109	[0.018, 0.448]
Moderator						
Interactional justice	0.098	0.096	[−0.091, 0.287]	0.126*	0.093	[−0.058, 0.309]
Interactive effect						
Inclusive leadership × Interactional justice	0.160**	0.061	[0.040, 0.281]	0.152*	0.060	[0.35, 0.270]
*R* ^2^	0.107**			0.0850**		
ΔR^2^	0.0250**			0.0240*		
F	3.312			2.569		
ΔF	6.837			6.530		

*N* = 258. *p ≤ 0.05. **p ≤ 0.01. ^1^ 1_male, 2_female; ^2^ 1_senior high school or below, 2_junior college, 3_college, 4_bachelor, 5_ postgraduate or above.

## 5 Discussion

It is critical to examine the impact of inclusive leadership on employee work behavior and how to better play the positive role of inclusive leadership (Guo et al., 2022; Jiang et al., 2022). Based on the social exchange theory, we explored how inclusive leadership affects employee promotive voice and prohibitive voice and whether the three dimensions of organizational justice perception affect the effectiveness of inclusive leadership. We found that inclusive leadership is positively related to employee promotive and prohibitive voice. The support and respect manifested by inclusive leaders enhance employees’ psychological safety, exert employees’ uniqueness, and encourage employees to give prohibitive advice. At the same time, inclusive leaders with openness and accessibility actively seek suggestions from subordinates and create a participatory circumstance to encourage employees to give promotive suggestions. In addition, the positive relationship between inclusive leadership and employee voice was strengthened at higher levels of organizational justice perception. Specifically, distributive justice and interactional justice strengthen the positive impact of inclusive leadership on employee promotive voice, and distributive justice, procedural justice, and interactional justice strengthen the positive impact of inclusive leadership on employee prohibitive voice.

### 5.1 Theoretical implications

The theoretical implications of this study can be discussed in several ways. First, we contribute to the literature of voice, by examining the impact of inclusive leadership on both employee promotive voice and prohibitive voice. Although the antecedents of employee voice have been widely explored, such as individual factors (e.g., personality, role definitions) and contextual factors (e.g., leader personality, relationship with supervisor; leadership style) (Detert and Burris, 2007; Morrison, 2011; Lin et al., 2017), the relationship-based leader perspectives that emphasize relationship and more process- and context-focused are ignored. Based on the social exchange theory, our study explores exchange patterns between inclusive leaders and employees, broadening the understanding of inclusive leadership as a predictor of voice and that it responds to the call for more studies exploring issues concerning promotive and prohibitive voice (Chamberlin et al., 2017). Besides, due to the findings of previous studies (Wong et al., 2010; Hsiung, 2012; Lin et al., 2017), we add humble leadership and authentic leadership as control variables. The results showed that the effect of inclusive leadership became more significant, which proved evidence that humble leadership and authentic leadership are positively related to employee voice. Thus, the results of our study highlight that leadership style plays an important role in an individual’s extra-role behaviors and ascertains the positive influence of inclusive leadership on employee voice.

Second, this study contributes to research on the inclusive leadership-employee voice relationship by furthering an understanding of the contextual conditions that can influence the relationship. Inclusive leadership has a significant impact on employee voice, and it is also a generic stance of the previous researchers (Qi and Liu, 2017; Guo et al., 2022; Jiang et al., 2022). Our results are intriguing when linked with the conditionality of employee organizational justice perception. We adopt the organizational justice view, and theoretically consider how the three dimensions of organizational justice affect employees’ perceptions of reciprocity and urge employees to provide more suggestions and concerns from a social exchange perspective. Our findings on the moderation role of employee perceived workplace justice enrich the knowledge of the contextual effectiveness of inclusive leadership. In addition, the findings demonstrated that distributed justice, procedural justice, and interactional justice play different roles in promoting the positive relationship between inclusive leadership and employee voice. Employee perception of workplace procedural justice did not moderate the positive relationship between inclusive leadership and employee promotive voice as we hypothesized.

### 5.2 Practical implications

This study has two practical implications. First, the result shows that inclusive leadership is positively related to employee promotive and prohibitive voice. This finding implies that an inclusive and “safe to speak up” environment helps encourage employees to speak out. For leaders, they need to show openness, accessibility, and availability in contact with followers (Carmeli et al., 2010). Through showing respect, recognition, and timely feedback, leaders can establish a high-quality relationship with subordinates (Nembhard and Edmondson, 2006; Hollander, 2012; Gallegos, 2013), alleviate their stress and strain, to encourage followers to speak up voluntarily. Enterprises can develop leadership training programs that emphasize self-development and expressions of inclusive behaviors to equip leaders with inclusive approaches and skills. At the same time, enterprises should shape an inclusive corporate culture and an inclusive organizational climate to develop and spread the concept of openness and fault tolerance within enterprises.

Second, the results of the moderating effect of workplace justice on the relationship between inclusive leadership and voice suggest that organizations and managers should strive to achieve and improve organizational justice. For distributive justice, organizations need to balance efficiency and equity in the distribution of salary, welfare, and promotions, and make timely adjustments to avoid excessive disparities (Choi et al., 2014). For procedural justice, organizations should ensure strict systems, fair standards, and transparent processes, and establish open information channels for employees to protect their right to know. For interactive justice, organizations should encourage leaders to actively interact with employees, improve the skills and methods of interaction between leaders and employees, and establish open and efficient communication channels for employees (Wong et al., 2006).

### 5.3 Limitations and future research directions

This study has several weaknesses. First, despite our time-lagged design in data collection, we cannot establish causality in the relationship between inclusive leadership and employee voice. Future research may adopt a longitudinal study to examine the possible dynamic relationship among the variables in this study. Second, workplace justice based on self-reporting was measured in the study. Hence, the employees’ perceptions of workplace justice are influenced by their subjective judgment. Future research may consider collecting other reported data to measure workplace justice. Third, although this study examined the direct relationship between inclusive leadership and employee promotive and prohibitive voice, we did not clarify the mechanism of the relationship. Further research can follow the social exchange approach and other approaches to explore mediators. Given the uncertain and potential outcome of voice (Liu et al., 2010), inclusive leadership may create a positive workplace climate, increase followers’ psychological security, and reduce power distance, thus enhancing their voice. Future studies could examine these potential mechanisms. Fourth, this study only examines employee perceptions of workplace justice as a moderator. Chamberlin et al. (2017) indicated, contextual factors such as workplace stressors (Morrison, 2011), positive or negative workplace climate may impact the effects of employee voice (George and Zhou, 2001; Lee et al., 2014; Zhang et al., 2014). Future studies can examine more individual, organizational, and societal mediators and moderators using a multi-level study design. Fifth, according to the results of previous studies (Wong et al., 2010; Hsiung, 2012; Lin et al., 2017), this study takes humble leadership and authentic leadership as control variables. Further study can consider relevant leadership when examining the relationship between the leader style and employee voice, to ensure the effectiveness and preciseness of the findings. Sixth, our research samples are from the Chinese construction industry, which may limit the generalizability of the findings. Considering the emphasis of a highly collectivism culture on global awareness, employees in such cultures may be more outspoken in proposing ideas and suggestions that benefit the organization (Zhang et al., 2016). Therefore, we call for future studies to test our research model with samples from different countries and analyze whether our findings can be replicated in individualistic cultures.

## Data availability statement

The raw data supporting the conclusions of this article will be made available by the authors, without undue reservation.

## Ethics statement

The studies involving humans were approved by Ethics Committee on Human Experimentation of Shandong University of Finance and Economics. The studies were conducted in accordance with the local legislation and institutional requirements. The participants provided their written informed consent to participate in this study.

## Author contributions

LQ: Writing – review & editing, Methodology, Resources, Writing – original draft. YX: Writing – original draft, Writing – review & editing. BL: Methodology, Resources, Writing – review & editing.

## References

[ref1] Abd El Majid E. Cohen A. (2015). The role of values and leadership style in developing OCB among Arab teachers in Israel. Leadership. Org. Dev. J. 36, 308–327. doi: 10.1108/LODJ-06-2013-0077

[ref2] Abu Nasra M. Heilbrunn S. (2016). Transformational leadership and organizational citizenship behavior in the Arab educational system in Israel: the impact of trust and job satisfaction. Educ. Manag. Adm. Lead. 44, 380–396. doi: 10.1177/1741143214549975

[ref3] Abuelhassan A. E. AlGassim A. (2022). How organizational justice in the hospitality industry influences proactive customer service performance through general self-efficacy. Int. J. Contemp. Hosp. M 34, 2579–2596. doi: 10.1108/IJCHM-10-2021-1238

[ref4] Adams J. S. (1963). Toward an understanding of inequity. J. Abnorm. Psychol. 67, 422–436. doi: 10.1037/h0040968, PMID: 14081885

[ref5] Adams J. S. (1965). Inequity in social exchange. Adv. Exp. Soc. Psychol. 2, 267–299. doi: 10.1016/S0065-2601(08)60108-2

[ref6] Adler P. S. Kwon S. W. (2002). Social capital: prospects for a new concept. Acad. Manag. Rev. 27, 17–40. doi: 10.2307/4134367, PMID: 36652326

[ref7] Agarwal U. A. (2014). Linking justice, trust and innovative work behaviour to work engagement. Pers. Rev. 43, 41–73. doi: 10.1108/PR-02-2012-0019

[ref8] Aiken L. S. West S. G. Reno R. R. (1991). Multiple regression: testing and interpreting interactions. New York: Sage.

[ref9] Ainscow M. Sandill A. (2010). Developing inclusive education systems: the role of organisational cultures and leadership. Int. J. Inclusive Educ. 14, 401–416. doi: 10.1080/13603110802504903, PMID: 34521658

[ref10] Alang T. Stanton P. Trau R. N. C. (2020). Exploring indigenous employee voice practice: perspectives from Vietnamese public sector organisations. Asia Pac. J. Humun. Resou. 58, 555–577. doi: 10.1111/1744-7941.12261

[ref11] Ambrose M. L. Schminke M. (2003). Organization structure as a moderator of the relationship between procedural justice, interactional justice, perceived organizational, support, and supervisory trust. J. Appl. Psychol. 88, 295–305. doi: 10.1037/0021-9010.88.2.295, PMID: 12731713

[ref12] Asghar Pourezzat A. Zeinali Someh P. (2009). The study of personnel and customers’ perception of organizational justice. Iran J. Manag. Stud. 2, 97–113. doi: 10.22059/ijms.2011.23393

[ref13] Avey J. B. Wernsing T. S. Palanski M. E. (2012). Exploring the process of ethical leadership: the mediating role of employee voice and psychological ownership. J. Bus. Ethics 107, 21–34. doi: 10.1007/s10551-012-1298-2

[ref14] Bies R. J. (1986). Interactional justice: communication criteria of fairness. Res. Negot. O 1, 43–55.

[ref15] Brockner J. Ackerman G. Greenberg J. Gelfand M. J. Francesco A. M. Chen Z. X.. (2001). Culture and procedural justice: the influence of power distance on reactions to voice. J. Exp. Soc. Psychol. 37, 300–315. doi: 10.1006/jesp.2000.1451

[ref16] Carmeli A. Reiter-Palmon R. Ziv E. (2010). Inclusive leadership and employee involvement in creative tasks in the workplace: the mediating role of psychological safety. Crea. Res. J. 22, 250–260. doi: 10.1080/10400419.2010.504654

[ref17] Carnevale J. B. Huang L. Crede M. Harms P. Uhl-Bien M. (2017). Leading to stimulate employees' ideas: a quantitative review of leader-member exchange, employee voice, creativity, and innovative behavior. Appl. Psychol. Int. Rev. 66, 517–552. doi: 10.1111/apps.12102

[ref18] Chamberlin M. Newton D. W. Lepine J. A. (2017). A meta-analysis of voice and its promotive and prohibitive forms: identification of key associations, distinctions, and future research directions. Pers. Psychol. 70, 11–71. doi: 10.1111/peps.12185

[ref19] Chen X. Cheng J. (2021). A review of the inclusive leadership research and its prospects from the vision of China. Sci. Res. Manage. 42, 174–181. doi: 10.19571/j.cnki.1000-2995.2021.10.020

[ref20] Chen S.-J. Wang M.-J. Lee S.-H. (2018). Transformational leadership and voice behaviors: the mediating effect of employee perceived meaningful work. Pers. Rev. 47, 694–708. doi: 10.1108/PR-01-2017-0016

[ref21] Chenevert D. Vandenberghe C. Tremblay M. (2015). Multiple sources of support, affective commitment, and citizenship behaviors the moderating role of passive leadership. Pers. Rev. 44, 69–90. doi: 10.1108/PR-08-2012-0144

[ref22] Choi B. K. Moon H. K. Ko W. Kim K. M. (2014). A cross-sectional study of the relationships between organizational justices and OCB roles of organizational identification and psychological contracts. Leadership. Org. Dev. J. 35, 530–554. doi: 10.1108/LODJ-08-2012-0103

[ref23] Chu T.-L. Lin W.-W. (2013). Uniqueness, integration or separation? Exploring the nature of creativity through creative writing by elementary school students in Taiwan. Educ. Psychol. 33, 582–595. doi: 10.1080/01443410.2013.821459, PMID: 37994749

[ref24] Cohen-Charash Y. Spector P. E. (2001). The role of justice in organizations: a meta-analysis. Organ. Behav. Hum. 86, 278–321. doi: 10.1006/obhd.2001.2958

[ref25] Colquitt J. A. Conlon D. E. Wesson M. J. Porter C. Ng K. Y. (2001). Justice at the millennium: a meta-analytic review of 25 years of organizational justice research. J. Appl. Psychol. 86, 425–445. doi: 10.1037/0021-9010.86.3.425, PMID: 11419803

[ref26] Cropanzano R. Bowen D. E. Gilliland S. W. (2007). The management of organizational justice. Acad. Manag. Perspect. 21, 34–48. doi: 10.5465/amp.2007.27895338, PMID: 37981332

[ref27] Cropanzano R. Byrne Z. S. Bobocel D. R. Rupp D. E. (2001). Self-enhancement biases, laboratory experiments, George Wilhelm Friedrich Hegel, and the increasingly crowded world of organizational justice. J. Vocat. Behav. 58, 260–272. doi: 10.1006/jvbe.2001.1798

[ref28] Cropanzano R. Mitchell M. S. (2005). Social exchange theory: an interdisciplinary review. J. Manage. 31, 874–900. doi: 10.1177/0149206305279602

[ref29] Detert J. R. Burris E. R. (2007). Leadership behavior and employee voice: is the door really open? Acad. Manag. J. 50, 869–884. doi: 10.5465/amj.2007.26279183

[ref30] Dollinger S. J. (2003). Need for uniqueness, need for cognition, and creativity. J. Creat. Behav. 37, 99–116. doi: 10.1002/j.2162-6057.2003.tb00828.x, PMID: 37290345

[ref31] Dooley R. S. Fryxell G. E. (1999). Attaining decision quality and commitment from dissent: the moderating effects of loyalty and competence in strategic decision-making teams. Acad. Manag. J. 42, 389–402. doi: 10.2307/257010

[ref32] Duc L. A. Tho N. D. (2023). Inclusive leadership and team innovation in retail services. Serv. Ind. J. 1-21, 1–21. doi: 10.1080/02642069.2023.2228205

[ref34] Ehrhart M. G. (2004). Leadership and procedural justice climate as antecedents of unit-level organizational citizenship behavior. Pers. Psychol. 57, 61–94. doi: 10.1111/j.1744-6570.2004.tb02484.x

[ref35] Emerson R. M. (1976). Social exchange theory. Annu. Rev. Sociol. 2, 335–362. doi: 10.1146/annurev.so.02.080176.002003, PMID: 37974096

[ref36] Farid H. Xiongying N. Raza J. Gul H. Hanif N. (2023). How and when organizational justice impact extra-role customer service: a social exchange perspective of thriving at work. Curr. Psychol. 42, 9743–9758. doi: 10.1007/s12144-021-02244-y

[ref37] Frey B. S. Homberg F. Osterloh M. (2013). Organizational control systems and pay-for-performance in the public service. Organ. Stud. 34, 949–972. doi: 10.1177/0170840613483655, PMID: 34010312

[ref38] Gallegos P. V. (2013). “The work of inclusive leadership” in Diversity at work: the practice of inclusion. eds. Ferdman B. M. Deane B. R. (San Francisco, CA: JosseyBass)

[ref39] George J. M. Zhou J. (2001). When openness to experience and conscientiousness are related to creative behavior: an interactional approach. J. Appl. Psychol. 86, 513–524. doi: 10.1037/0021-9010.86.3.513, PMID: 11419810

[ref40] Gouldner A. W. (1960). The norm of reciprocity: a preliminary statement. Am. Sociol. Rev. 25, 161–178. doi: 10.2307/2092623

[ref41] Graham J. W. Van Dyne L. (2006). Gathering information and exercising influence: two forms of civic virtue organizational citizenship behavior. Emp. Responsib. Rig. 18, 89–109. doi: 10.1007/s10672-006-9007-x

[ref42] Greenberg J. (1990). Organizational justice: yesterday, today, and tomorrow. J. Manage. 16, 399–432. doi: 10.1177/014920639001600208

[ref43] Guh W.-Y. Lin S.-P. Fan C.-J. Yang C.-F. (2013). Effects of organizational justice on organizational citizenship Behaviors: mediating effects of institutional trust and affective commitment. Psychol. Rep. 112, 818–834. doi: 10.2466/01.21.PR0.112.3.818-834, PMID: 24245075

[ref44] Guo Y. Zhu Y. Zhang L. (2022). Inclusive leadership, leader identification and employee voice behavior: the moderating role of power distance. Curr. Psychol. 41, 1301–1310. doi: 10.1007/s12144-020-00647-x

[ref45] Guzman F. A. Espejo A. (2019). Introducing changes at work: how voice behavior relates to management innovation. J. Organ. Behav. 40, 73–90. doi: 10.1002/job.2319

[ref46] Harris T. B. Li N. Kirkman B. L. (2014). Leader-member exchange (LMX) in context: how LMX differentiation and LMX relational separation attenuate LMX's influence on OCB and turnover intention. Leadership. Quart. 25, 314–328. doi: 10.1016/j.leaqua.2013.09.001

[ref47] Hartman S. J. Yrle A. C. Galle W. P. (1999). Procedural and distributive justice: examining equity in a university setting. J. Bus. Ethics 20, 337–351. doi: 10.1023/A:1006102216883

[ref48] Hayes A. F. (2017). Introduction to mediation, moderation, and conditional process analysis: a regression-based approach. New York: Guilford Publications.

[ref49] He W. Fehr R. Yam K. C. Long L.-R. Hao P. (2017). Interactional justice, leader-member exchange, and employee performance: examining the moderating role of justice differentiation. J. Organ. Behav. 38, 537–557. doi: 10.1002/job.2133

[ref50] Hirak R. Peng A. C. Carmeli A. Schaubroeck J. M. (2012). Linking leader inclusiveness to work unit performance: the importance of psychological safety and learning from failures. Leadership. Quart. 23, 107–117. doi: 10.1016/j.leaqua.2011.11.009

[ref51] Hoffman B. J. Blair C. A. Meriac J. P. Woehr D. J. (2007). Expanding the criterion domain? A quantitative review of the OCB literature. J. Appl. Psychol. 92, 555–566. doi: 10.1037/0021-9010.92.2.555, PMID: 17371100

[ref52] Holland P. Cooper B. Sheehan C. (2016). Employee voice, supervisor support, and engagement: the mediating role of trust. Hum. Resour. Manage. 56, 915–929. doi: 10.1002/hrm.21809

[ref53] Hollander E. (2012). Inclusive leadership: the essential leader-follower relationship. Abingdon-on-Thames: Routledge.

[ref54] Homans G. C. (1958). Social behavior as exchange. Am. J. Sociol. 63, 597–606. doi: 10.1086/222355, PMID: 37974096

[ref55] Hon A. H. Y. Lu L. (2010). The mediating role of trust between expatriate procedural justice and employee outcomes in Chinese hotel industry. Int. J. Hosp. Manag. 29, 669–676. doi: 10.1016/j.ijhm.2010.01.002

[ref56] Hopkins S. M. Weathington B. L. (2006). The relationships between justice perceptions, trust, and employee attitudes in a downsized organization. J. Psychol. 140, 477–498. doi: 10.3200/JRLP.140.5.477-498, PMID: 17066753

[ref57] Howell T. M. Harrison D. A. Burris E. R. Detert J. R. (2015). Who gets credit for input? Demographic and structural status cues in voice recognition. J. Appl. Psychol. 100, 1765–1784. doi: 10.1037/apl0000025, PMID: 25915784

[ref58] Hsiung H.-H. (2012). Authentic leadership and employee voice behavior: a multi-level psychological process. J. Bus. Ethics 107, 349–361. doi: 10.1007/s10551-011-1043-2

[ref59] Javed B. Naqvi S. M. M. R. Khan A. K. Arjoon S. Tayyeb H. H. (2017). Impact of inclusive leadership on innovative work behavior: the role of psychological safety. J. Manage. Organ. 25, 117–136. doi: 10.1017/jmo.2017.3

[ref60] Jiang J. Ding W. Wang R. Li S. (2022). Inclusive leadership and employees' voice behavior: a moderated mediation model. Curr. Psychol. 41, 6395–6405. doi: 10.1007/s12144-020-01139-8

[ref61] Jin M. Lee J. Lee M. (2017). Does leadership matter in diversity management? Assessing the relative impact of diversity policy and inclusive leadership in the public sector. Leadership. Org. Dev. J. 38, 303–319. doi: 10.1108/LODJ-07-2015-0151

[ref62] Kim W. C. Mauborgne R. (1997). Fair process: managing in the knowledge economy. Harvard Bus. Rev. 75, 65–75. 10168337

[ref63] Konovsky M. A. (2000). Understanding procedural justice and its impact on business organizations. J. Manage. 26, 463–488. doi: 10.1177/014920630002600305

[ref64] Kwon B. Farndale E. Park J. G. (2016). Employee voice and work engagement: macro, meso, and micro-level drivers of convergence? Hum. Resour. Manage R 26, 327–337. doi: 10.1016/j.hrmr.2016.04.005

[ref65] Lam C. F. Mayer D. M. (2014). When do employees speak up for their customers? A model of voice in a customer service context. Pers. Psychol. 67, 637–666. doi: 10.1111/peps.12050

[ref66] Lavelle J. J. (2010). What motivates OCB? Insights from the volunteerism literature. J. Organ. Behav. 31, 918–923. doi: 10.1002/job.644

[ref67] Lee D. Choi Y. Youn S. Chun J. U. (2017). Ethical leadership and employee moral voice: the mediating role of moral efficacy and the moderating role of leader-follower value congruence. J. Bus. Ethics 141, 47–57. doi: 10.1007/s10551-015-2689-y

[ref68] Lee G. L. Diefendorff J. M. Kim T.-Y. Bian L. (2014). Personality and participative climate: antecedents of distinct voice behaviors. Hum. Perform. 27, 25–43. doi: 10.1080/08959285.2013.854363

[ref69] Li A. N. Liao H. Tangirala S. Firth B. M. (2017). The content of the message matters: the differential effects of promotive and prohibitive team voice on team productivity and safety performance gains. J. Appl. Psychol. 102, 1259–1270. doi: 10.1037/apl0000215, PMID: 28358532

[ref70] Li Y. Sun J.-M. (2015). Traditional Chinese leadership and employee voice behavior: a cross-level examination. Leadership. Quart. 26, 172–189. doi: 10.1016/j.leaqua.2014.08.001

[ref71] Li A. N. Tangirala S. (2021). How voice emerges and develops in newly formed supervisor–employee dyads. Acad. Manag. J. 64, 614–642. doi: 10.5465/amj.2018.0961

[ref72] Liang J. Farh C. I. C. Farh J.-L. (2012). Psychological antecedents of promotive and prohibitive voice: a two-wave examination. Acad. Manag. J. 55, 71–92. doi: 10.5465/amj.2010.0176

[ref73] Lin X. Chen Z. X. Tse H. H. M. Wei W. Ma C. (2017). Why and when employees like to speak up more under humble leaders? The roles of personal sense of power and power distance. J. Bus. Ethics 158, 937–950. doi: 10.1007/s10551-017-3704-2

[ref74] Lind E. A. (2001). “Fairness heuristic theory: justice judgments as pivotal cognitions in organizational relations” in Advances in organization justice. eds. Greenberg J. Cropanzano R. (Stanford: Stanford University Press)

[ref75] Lind E. Tyler T. (1988). Critical issues in social justice. Springer: New York.

[ref76] Lipponen J. Olkkonen M.-E. Myyry L. (2004). Personal value orientation as a moderator in the relationships between perceived organizational justice and its hypothesized consequences. Soc. Justice Res 17, 275–292. doi: 10.1023/B:SORE.0000041294.68845.0f

[ref77] Liu F. Chow I. H.-S. Gong Y. Huang M. (2019). Affiliative and aggressive humor in leadership and their effects on employee voice: a serial mediation model. Rev. Manag. Sci. 14, 1321–1339. doi: 10.1007/s11846-019-00334-7

[ref78] Liu W. Zhu R. Yang Y. (2010). I warn you because I like you: voice behavior, employee identifications, and transformational leadership. Leadership. Quart. 21, 189–202. doi: 10.1016/j.leaqua.2009.10.014

[ref79] Mackenzie S. B. Podsakoff P. M. Podsakoff N. P. (2011). Challenge oriented organizational citizenship behaviors and organizational effectiveness: do challenge-oriented behaviors really have an impact on the organization's bottom line? Pers. Psychol. 64, 559–592. doi: 10.1111/j.1744-6570.2011.01219.x

[ref80] Milliken F. J. Morrison E. W. Hewlin P. F. (2003). An exploratory study of employee silence: issues that employees don't communicate upward and why. J. Manage. Stud. 40, 1453–1476. doi: 10.1111/1467-6486.00387

[ref81] Morrison E. W. (2011). Employee voice behavior: integration and directions for future research. Acad. Manag. Ann. 5, 373–412. doi: 10.5465/19416520.2011.574506, PMID: 35431247

[ref82] Nahrgang J. D. Morgeson F. P. Hofmann D. A. (2011). Safety at work: a meta-analytic investigation of the link between job demands, job resources, burnout, engagement, and safety outcomes. J. Appl. Psychol. 96, 71–94. doi: 10.1037/a0021484, PMID: 21171732

[ref83] Neider L. L. Schriesheim C. A. (2011). The authentic leadership inventory (ALI): development and empirical tests. Leadership. Quart. 22, 1146–1164. doi: 10.1016/j.leaqua.2011.09.008

[ref84] Nembhard I. M. Edmondson A. C. (2006). Making it safe: the effects of leader inclusiveness and professional status on psychological safety and improvement efforts in health care teams. J. Organ. Behav. 27, 941–966. doi: 10.1002/job.413

[ref85] Ng T. W. H. Feldman D. C. (2011). Employee voice behavior: a meta-analytic test of the conservation of resources framework. J. Organ. Behav. 33, 216–234. doi: 10.1002/job.754

[ref86] Niehoff B. P. Moorman R. H. (1993). Justice as a mediator of the relationship between methods of monitoring and organizational citizenship behavior. Acad. Manag. J. 36, 527–556. doi: 10.2307/256591

[ref87] Nohe C. Hertel G. (2017). Transformational leadership and organizational citizenship behavior: a meta-analytic test of underlying mechanisms. Front. Psychol. 8:1364. doi: 10.3389/fpsyg.2017.01364, PMID: 28848478 PMC5554340

[ref88] Owens B. P. Johnson M. D. Mitchell T. R. (2013). Expressed humility in organizations: implications for performance, teams, and leadership. Organ. Sci. 24, 1517–1538. doi: 10.1287/orsc.1120.0795

[ref89] Pichler S. Varma A. Michel J. S. Levy P. E. Budhwar P. S. Sharma A. (2015). Leader-member exchange, group- and individual-level procedural justice and reactions to performance appraisals. Hum. Resour. Manage. 55, 871–883. doi: 10.1002/hrm.21724

[ref90] Pinder C. C. Harlos K. P. (2001). “Employee silence: quiescence and acquiescence as responses to perceived injustice” in Research in personnel and human resources management. eds. Rowland K. M. Ferris G. R. (Bingley: Emerald Group Publishing Limited)

[ref91] Podsakoff P. M. MacKenzie S. B. Lee J. Y. Podsakoff N. P. (2003). Common method biases in behavioral research: a critical review of the literature and recommended remedies. J. Appl. Psychol. 88, 879–903. doi: 10.1037/0021-9010.88.5.879, PMID: 14516251

[ref92] Premeaux S. F. Bedeian A. G. (2003). Breaking the silence: the moderating effects of self-monitoring in predicting speaking up in the workplace. J. Manage. Stud. 40, 1537–1562. doi: 10.1111/1467-6486.00390

[ref93] Qi L. Liu B. (2017). Effects of inclusive leadership on employee voice behavior and team performance: the mediating role of caring ethical climate. Front. Commun. 2:8. doi: 10.3389/fcomm.2017.00008

[ref94] Randel A. E. Galvin B. M. Shore L. M. Ehrhart K. H. Chung B. G. Dean M. A.. (2018). Inclusive leadership: realizing positive outcomes through belongingness and being valued for uniqueness. Hum. Resour. Manage. R 28, 190–203. doi: 10.1016/j.hrmr.2017.07.002

[ref95] Santos M. Luna M. Reyes D. L. Traylor A. Lacerenza C. N. Salas E. (2022). How to be an inclusive leader for gender-diverse teams. Organ. Dyn. 51:100914. doi: 10.1016/j.orgdyn.2022.100914

[ref96] Settoon R. P. Bennett N. Liden R. C. (1996). Social exchange in organizations: perceived organizational support, leader–member exchange, and employee reciprocity. J. Appl. Psychol. 81, 219–227. doi: 10.1037/0021-9010.81.3.219, PMID: 23834589

[ref97] Shoaib S. Baruch Y. (2017). Deviant behavior in a moderated-mediation framework of incentives, organizational justice perception, and reward expectancy. J. Bus. Ethics 157, 617–633. doi: 10.1007/s10551-017-3651-y

[ref98] Tangirala S. Ramanujam R. (2008). Exploring nonlinearity in employee voice: the effects of personal control and organizational identification. Acad. Manag. J. 51, 1189–1203. doi: 10.5465/amj.2008.35732719

[ref99] Thibaut J. W. Walker L. (1975). Procedural justice: a psychological analysis. Duke Law J. 1977:1289. doi: 10.2307/1371953

[ref100] Thierry H. (2002). Enhancing performance through pay and reward systems. Psychol. Manag. Individ. Performan. doi: 10.1002/0470013419.ch16

[ref101] Tremblay M. Cloutier J. Simard G. Chenevert D. Vandenberghe C. (2010). The role of HRM practices, procedural justice, organizational support and trust in organizational commitment and in-role and extra-role performance. Int. J. Hum. Resour. Manage. 21, 405–433. doi: 10.1080/09585190903549056

[ref102] Tyler T. R. Lind E. A. (1992). A relational model of authority in groups. Adv. Exp. Soc. Psychol. 25, 115–191. doi: 10.1016/s0065-2601(08)60283-x

[ref33] Van Dyne L. Ang S. Botero I. C. (2003). Conceptualizing employee silence and employee voice as multidimensional constructs. J. Manag. Stud. 40, 1359–1392. doi: 10.1111/1467-6486.00384

[ref103] Van Dyne L. LePine J. A. (1998). Helping and voice extra-role behaviors: evidence of construct and predictive validity. Acad. Manag. J. 41, 108–119. doi: 10.2307/256902

[ref104] Walster E. Berscheid E. (1973). New directions in equity research. J. Pers. Soc. Psychol. 25, 151–176. doi: 10.1037/h0033967, PMID: 37990347

[ref105] Walumbwa F. O. Schaubroeck J. (2009). Leader personality traits and employee voice behavior: mediating roles of ethical leadership and work group psychological safety. J. Appl. Psychol. 94, 1275–1286. doi: 10.1037/a0015848, PMID: 19702370

[ref106] Wayne S. J. Shore L. M. Liden R. C. (1997). Perceived organizational support and leader-member exchange: a social exchange perspective. Acad. Manag. J. 40, 82–111. doi: 10.2307/257021, PMID: 37333594

[ref107] Wei X. Zhang Z.-X. Chen X.-P. (2015). I will speak up if my voice is socially desirable: a moderated mediating process of promotive versus prohibitive voice. J. Appl. Psychol. 100, 1641–1652. doi: 10.1037/a0039046, PMID: 25844927

[ref108] Wong C. A. Laschinger H. K. S. Cummings G. G. (2010). Authentic leadership and nurses' voice behaviour and perceptions of care quality. J. Nurs. Manage. 18, 889–900. doi: 10.1111/j.1365-2834.2010.01113.x, PMID: 21073563

[ref109] Wong Y.-T. Ngo H.-Y. Wong C.-S. (2006). Perceived organizational justice, trust, and OCB: a study of Chinese workers in joint ventures and state-owned enterprises. J. World Bus. 41, 344–355. doi: 10.1016/j.jwb.2006.08.003

[ref110] Ye Q. Wang D. Guo W. (2019). Inclusive leadership and team innovation: the role of team voice and performance pressure. Eur. Manag. J. 37, 468–480. doi: 10.1016/j.emj.2019.01.006

[ref111] Zhang S. Chen G. Chen X.-P. Liu D. Johnson M. D. (2014). Relational versus collective identification within workgroups: conceptualization, measurement development, and nomological network building. J. Manage. 40, 1700–1731. doi: 10.1177/0149206312439421

[ref112] Zhang L. Yang F. Gu Y. (2016). Inclusive leadership: conception, measurement and relationships to related variables. Adv. Psychol. Sci. 24:1467. doi: 10.3724/SP.J.1042.2016.01467

